# Exploiting the Achilles’ heel of cancer: disrupting glutamine metabolism for effective cancer treatment

**DOI:** 10.3389/fphar.2024.1345522

**Published:** 2024-03-06

**Authors:** Yuxin Fan, Han Xue, Zhimin Li, Mingge Huo, Hongxia Gao, Xingang Guan

**Affiliations:** ^1^ Department of Clinical Laboratory Diagnostics, School of Medical Technology, Beihua University, Jilin City, China; ^2^ Department of Basic Medicine, Medical School, Taizhou University, Taizhou, Zhejiang Province, China

**Keywords:** glutamine, glutamine metabolism, glutamine uptake, cancer therapy, combination therapy

## Abstract

Cancer cells have adapted to rapid tumor growth and evade immune attack by reprogramming their metabolic pathways. Glutamine is an important nitrogen resource for synthesizing amino acids and nucleotides and an important carbon source in the tricarboxylic acid (TCA) cycle and lipid biosynthesis pathway. In this review, we summarize the significant role of glutamine metabolism in tumor development and highlight the vulnerabilities of targeting glutamine metabolism for effective therapy. In particular, we review the reported drugs targeting glutaminase and glutamine uptake for efficient cancer treatment. Moreover, we discuss the current clinical test about targeting glutamine metabolism and the prospective direction of drug development.

## 1 Introduction

Cancer cells have adapted to rapid tumor growth in harsh environments by reprogramming their metabolic pathways (Martínez-Reyes and Chandel, 2021; Xu et al., 2023). Cell metabolism in cancers is extremely active to obtain sufficient nutrients to meet the high demands of energy and resources (Faubert et al., 2020; Zhao et al., 2023). For example, cancer cells produce lactate through glycolysis to satisfy their energy needs even in the presence of oxygen, known as the Warburg effect (Ward and Thompson, 2012). Aerobic glycolysis provides cancer cells with sufficient energy resources and necessary macromolecules for cell growth and proliferation (Vander Heiden et al., 2009).

Recent advances have indicated that glutamine metabolism also plays a key role in tumor development and metastasis (Jain et al., 2012; Kodama et al., 2020; Oh et al., 2020). As the most abundant non-essential amino acid in the blood and muscle, glutamine can provide energy and a precursor for the biosynthesis of nucleotides, amino acids, and lipids (Tong et al., 2009; Altman et al., 2016; Choi and Coloff, 2019; Cooper et al., 2023; Jin et al., 2023). In addition, the intracellular glutamine can maintain redox homeostasis through many sophisticated mechanisms (Son et al., 2013; Koppula et al., 2018; Jaganjac et al., 2020; Ying et al., 2021). The proliferation of cancer cells highly depends on the extracellular glutamine supply, termed “glutamine addiction” (Wise and Thompson, 2010). Besides cancer cells, glutamine also participates in the immune cells’ proliferation, promoting the secretion of pro-inflammatory cytokines (Vergadi et al., 2017; Cruzat et al., 2018). Given the essential role of glutamine supply in cancer cell proliferation and immune cell phenotypes, developing novel antitumor drugs targeting glutamine metabolism has shown promising therapeutic effects in various mouse cancer models and preclinical tests (Oh et al., 2020; Sharma et al., 2020; Edwards et al., 2021; Lee et al., 2022).

In this review, we summarize the significant role of glutamine metabolism in tumor development and highlight the vulnerabilities of targeting glutamine metabolism for effective therapy. In particular, we review the reported drugs targeting glutamine metabolism for cancer treatment. This review aims to help understand the latest advances in novel drugs targeting glutamine metabolism and combination therapy strategies for enhanced cancer therapy.

## 2 Glutamine Metabolism in cancer Cells

**FIGURE 1:**
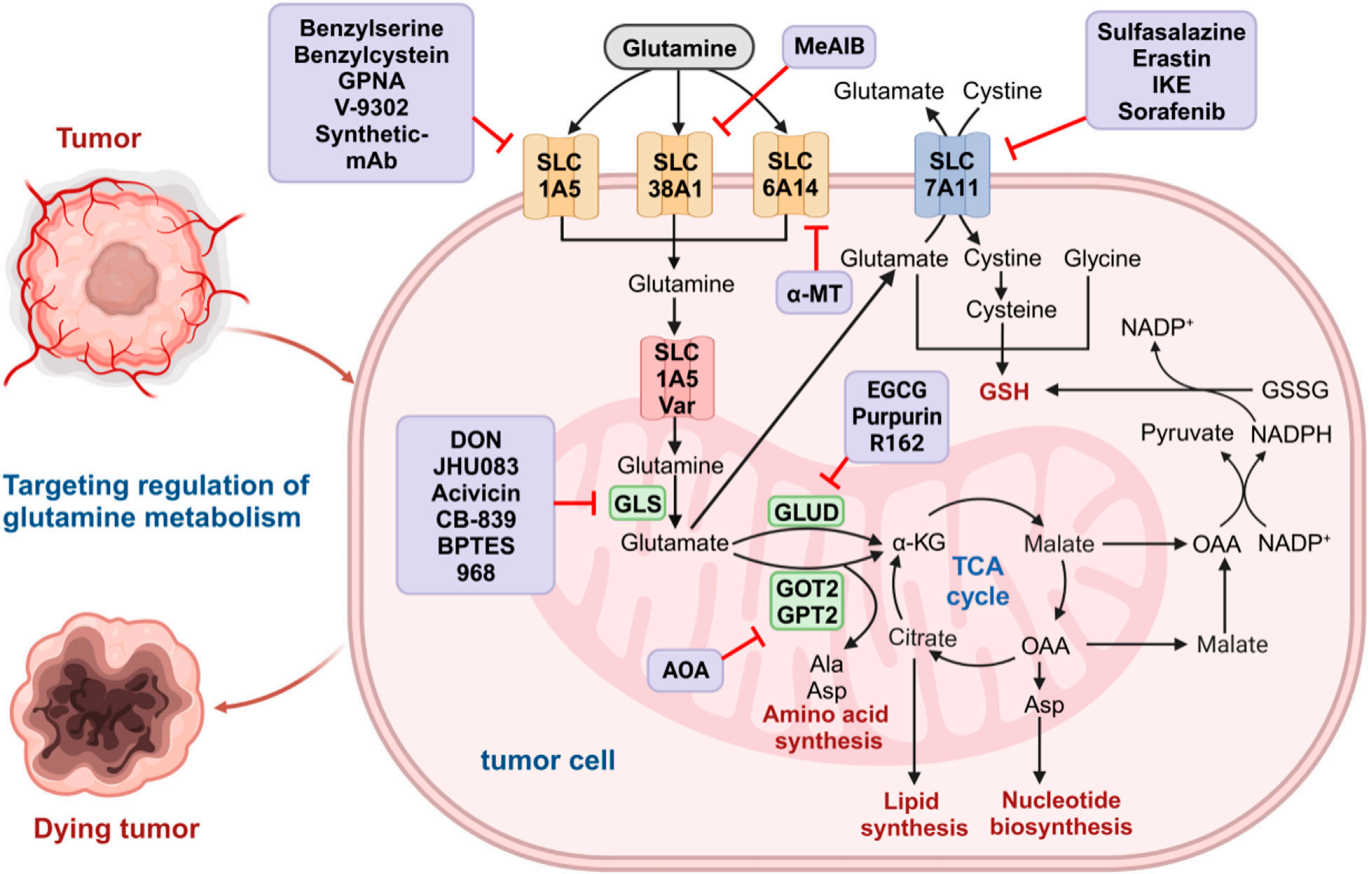
Glutamine metabolism pathways in cancer cells. Glutamine is transported to the cytoplasm by glutamine transporters on the cell membrane (SLC1A5, SLC38A1, and SLC6A14) and then to the mitochondria via SLC1A5 variants. In the mitochondrial matrix, glutamine is converted to glutamate by GLS. The resulting glutamate is converted into α-KG by GLUD1, GOT2, and GPT2 to enter the TCA cycle and further participate in the biosynthesis of amino acids, nucleotides, and lipids. Malic acid produced by the TCA cycle crosses the mitochondrial membrane into the cytoplasm and is oxidized by malate dehydrogenase to produce OAA. The OAA can be converted to aspartic acid and transported out of the cytoplasm, where malic acid and further OAA are formed. The OAA in the cytoplasm is converted to pyruvate by malidase and is used to produce NADPH, which is involved in maintaining redox homeostasis in tumor cells. Glutamate produced by glutamine in mitochondria can escape to the cytoplasm and be transported by SLC7A11 on the cell membrane to exchange cystine, further forming cysteine and participating in *de novo* synthesis of GSH. The medium purple box show the glutamine metabolic inhibitors at each step. GLS, glutaminase; α-KG, α-ketoglutarate; GLUD1, glutamate dehydrogenase 1; GOT, glutamate oxaloacetate transaminase; GPT, glutamate pyruvate transaminase; TCA, tricarboxylic acid; OAA, oxaloacetic acid; NADPH, nicotinamide adenine dinucleotide phosphate; GSSG, oxidized glutathione; GSH, oxidized glutathione.

Glutamine is an important nitrogen resource for synthesizing amino acids and nucleotides and an important carbon source in the TCA cycle and lipid biosynthesis pathway (Figure 1) (Zhang et al., 2017; Cooper et al., 2023). Due to the rapid consumption of glutamine, cancer cells cannot meet the huge demand for glutamine through self-synthesis (Wise and Thompson, 2010). To achieve sufficient glutamine, cancer cells must uptake glutamine from the extracellular environment through specific transporters on the cell membrane, such as the alanine-serine-cysteine transporter 2 (ASCT2), also known as SLCA15 (Scalise et al., 2017). The intracellular glutamine is metabolized into glutamate and ammonia by the mitochondria’s enzyme glutaminase (GLS) (Still and Yuneva, 2017). Glutamate is then converted to α-ketoglutarate (α-KG) by mitochondrial glutamate dehydrogenase (GLUD) (Altman et al., 2016; Zhang et al., 2017). α-KG enters the TCA cycle to provide energy and contributes to acetyl-CoA production. Acetyl coenzyme A is transported to the cytoplasm through the citric acid-isocitric acid cycle, where it participates in fatty acid synthesis (Mullen et al., 2014; Pavlova and Thompson, 2016; Zhang et al., 2017; Vanhove et al., 2019). The generated glutamate can also be used to produce other non-essential amino acids through transaminase activity (Altman et al., 2016; Choi and Coloff, 2019; Jin et al., 2023).

In addition, glutamine plays an important role in maintaining the redox homeostasis of tumor cells. As one of the important amino acids, glutamine participates in the synthesis of glutathione (GSH). Glutamate can undergo polymerization with cysteine to form gamma-glutamylcysteine, which is further condensed with glycine to produce GSH (Lushchak, 2012; Lukey et al., 2016). Malate generated in the TCA cycle can be converted to nicotinamide adenine dinucleotide phosphate (NADPH) under the catalysis of the malic enzyme (Son et al., 2013; Ying et al., 2021). NADPH can reduce oxidized glutathione (GSSG) to GSH and convert cystine to cysteine, participating in the *de novo* synthesis of GSH (Jaganjac et al., 2020). GSH is an important endogenous antioxidant that can eliminate oxidative damage caused by excessive reactive oxygen species (ROS). Therefore, disrupting the redox homeostasis of cancer cells by inhibiting glutamine metabolism may be an effective strategy for cancer treatment (Byun et al., 2020). Glutamine can also suppress tumor growth by inhibiting cell autophagy through mTOR pathway activation and ROS elimination (Dewaele et al., 2010; Nazio et al., 2013; Li Q. et al., 2022). However, the ammonia produced in glutamine metabolism can promote cell autophagy, contributing to tumor growth (Eng et al., 2010; Cheong et al., 2012). The concrete role of glutamine in regulating cell autophagy is still not fully understood and requires further research.

Besides tumor cells, immune cells also rely on glutamine to maintain their proliferation and activation (Bodineau et al., 2022). In macrophages, glutamine can regulate the synthesis and secretion of pro-inflammatory cytokines such as tumor necrosis factor-α (TNF-α), interleukin-1 (IL-1), and interleukin-6 (IL-6) (Vergadi et al., 2017; Cruzat et al., 2018). It can also activate extracellular signal-regulated kinase (ERK) and c-Jun N-terminal kinase (JNK), upregulating the proliferation-related gene expression in immune cells. The suitable glutamine concentration promotes the expression of surface markers and the production of cytokines (IFN-γ, TNF-α) in lymphocyte cells (Cruzat et al., 2018). In a mouse triple-negative breast cancer (TNBC) model, inhibiting glutamine uptake in tumor cells but not T cells can trigger superior T cell responses, leading to diminished tumor growth (Edwards et al., 2021).

## 3 Drugs targeting glutamine metabolism for cancer treatment

Given the significant reliance of cancer cells on glutamine, developing novel drugs targeting this vulnerability has emerged as an effective strategy for cancer treatment. The reported drugs mainly aim at glutamine transporters, glutaminase, aminotransferases, GSH, and NADPH synthesis (Table 1) (Choi and Park, 2018; Li L. et al., 2019). In mouse cancer models, these drugs alone or combined with other treatments exhibit remarkable therapeutic effects (Gross et al., 2014; Gregory et al., 2019; Edwards et al., 2021; Li Q. et al., 2022).

**TABLE 1:** Pharmacological strategies for inhibiting glutamine metabolism in cancer cells.

Inhibitor	Mechanism	Limitation	Tumor type	Application and effect	Reference
BPTES	GLS1 inhibitor	Low bioavailability, poor metabolic stability, and low solubility	Glioma, Chondrosarcoma, AML	Interfere with the metabolism and growth of IDH mutant tumor cells	Seltzer et al. (2010), Emadi et al. (2014), Zhang et al. (2022)
CB-839	GLS1 inhibitor	Higher calculated logP, and lower lipophilic efficiency	TNBC	Destroy cell redox homeostasis and enhance the cytotoxicity of platinum	Hong et al. (2022)
				61% of tumor growth inhibited	Gross et al. (2014)
			Melanoma	Activate antigen-specific T cells and increase tumor infiltration of effector T cells	Varghese et al. (2021)
			RCC	Telaglenastat plus everolimus (TelaE) enhanced the survival rate	Lee et al. (2022)
			AML、MM	Induce the accumulation of mitoROS and damage energy metabolism	Gregory et al. (2019), Timofeeva et al. (2023)
			Colorectal Cancer	Increase the level of ROS in tumor cells and enhance the inhibitory effect of thymidylate synthase	Zhao et al. (2020)
				Inhibit tumor proliferation, inhibit mTOR-mediated survival signal transduction, and promote tumor cell apoptosis and necrosis	Cohen et al. (2020)
Compound 968	GAC inhibitor	Potential toxic side effects	Ovarian Cancer	Enhance the activation and function of T cells and promote the infiltration of T cells	Wang et al. (2021)
			Endometrial Cancer, Ovarian Cancer	Induce G1 cell cycle arrest, apoptosis, and cell stress, and inhibit the growth of tumor cells	Yuan et al. (2016), Guo et al. (2023)
			HCC	Block the reprogramming metabolism of tumor cells, and synergize dihydroartemisinin to increase the intracellular ROS level	Wang et al. (2016)
DON	Glutamine antagonist	Severe systemic toxicity	PDAC	Target hexosamine biosynthesis pathway, and then remodel tumor extracellular matrix	Sharma et al. (2020)
JHU083	Glutamine antagonist	Low selectivity, potential toxic effects	TNBC	Inhibit the production and recruitment of MDSC, promote the ratio of anti-tumor inflammatory TAM, and reduce the level of immunosuppressive cells	Oh et al. (2020)
			Medulloblastoma	It has strong brain penetration and significantly improves the survival rate of mice	Hanaford et al. (2019)
			Glioma	Prolong survival in the intracranial glioma model	Yamashita et al. (2021)
GPNA	Glutamine transporter (ASCT2)inhibitor	Low selectivity and high toxicity	Non-small cell lung cancer (NSCLC)	Induce autophagy and apoptosis and suppress tumor growth in NSCLC xenografts	Hassanein et al. (2015)
			Gastric Cancer	Inhibit tumor growth and enhance the therapeutic effect of cetuximab on gastric cancer	Ma et al. (2021)
			Prostate Cancer	Reduce the basal oxygen consumption rate (OCR) and cellular lipid levels	Wang et al. (2015a)
			HCC	Inhibit cholesterol synthesis and enhance the antitumor effect	Kong et al. (2023)
V-9302	Glutamine transporter (ASCT2)inhibitor	Poor water solubility, rapid clearance in the body, poor tissue permeability	Breast cancer	Promote ROS-induced B7H3 autophagy and enhance the antitumor effect of anti-PD-1 immunotherapy	Li et al. (2022a)
				Inhibit glutamine uptake by TNBC cells and improve redox balance in T cells	Edwards et al. (2021)
			RCC	Promote cell aging and inhibit tumor growth, invasion, and migration	Kawakami et al. (2022)
			Colon Cancer	Upregulate the expression of PD-L1 and Fas and improve the immunotherapy effect of anti-PD-L1	Byun et al. (2020)
			Ovarian Cancer	Inhibit mTORC1/S6K signal pathway and sensitize SKOV3-TR cells to paclitaxel	Kim et al. (2022)
Benzylserine/benzylcysteine derivatives	Glutamine transporter (ASCT2)inhibitor	Low specificity, high dose requirement	Melanoma, Breast Cancer	Promote the decreased cell viability and cell cycle progression	Wang et al. (2014), van Geldermalsen et al. (2018)
mAb	Glutamine transporter (ASCT2)inhibitor	High price, poor penetration	Colorectal Cancer	KM4008, KM4012, and KM4018 inhibit cell proliferation in a dose-dependent manner	Suzuki et al. (2017)
				Reduce glutamine transport in SW1116 and HCT116 colon cancer cells and suppress tumor growth *in vivo*	Hara et al. (2020)
			Gastric Cancer	Inhibit tumor growth *in vivo* by ADCC.	Osanai-Sasakawa et al. (2018)
EGCG	GLUD inhibitor	Poor bioavailability and low stability	Breast, Prostate, Cervical, and Pancreatic Cancer	Inhibit tumor growth and prevent metastasis	Stuart et al. (2006), Shankar et al. (2008), Wang et al. (2018), Romano and Martel (2021)
R162	GLUD inhibitor	Potential toxic side effects	Breast Cancer, Lung Cancer, and AML	Disrupting the redox homeostasis of cancer cells	Jin et al. (2015), Jin et al. (2018)
AOA	GOT2 and GPT2 inhibitor	Potential toxic side effects	Breast Cancer	Endoplasmic reticulum stress-induced tumor cell growth inhibition and apoptosis	Korangath et al. (2015)
			Colon Cancer	Inhibition of PIK3CA mutation-driven tumorigenesis	Hao et al. (2016)

### 3.1 Glutaminase inhibitors

Glutaminases (GLS) are overexpressed in many cancer cells, making them a diagnosis biomarker and therapeutic target for glutamine addiction (Yang W.-H. et al., 2021). GLS in the mitochondria catalyzes the breakdown of glutamine to produce glutamate and ammonia (Van Den Heuvel et al., 2012; Lukey et al., 2013). There are two subtypes of glutaminase in the human body: kidney-type glutaminase (GLS1) and liver-type glutaminase (GLS2) (Wang et al., 2010; Ferreira et al., 2013).

**FIGURE 2:**
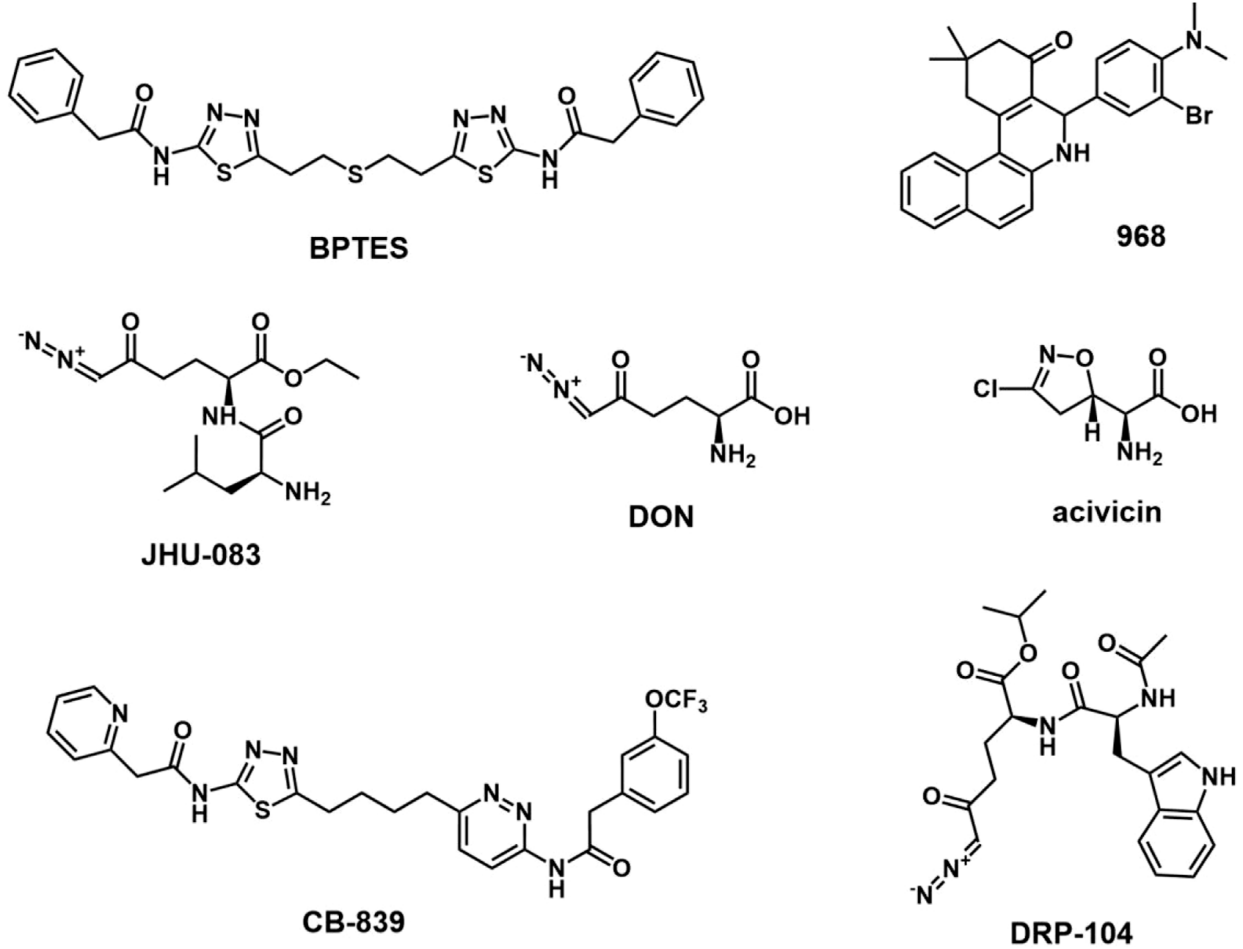
Chemical structure of inhibitors targeting glutaminase.

GLS1, encoded by the GLS gene on human chromosome 2, is inactive in the dimer state and activated in the phosphorylation state which it assembled into tetramers. There are two splicing variants of GLS1: a long form of renal glutaminase (KGA) and a short form of glutaminase C (GAC) (Wang et al., 2010; Ferreira et al., 2013). The C-terminal difference between the two variants makes GAC more specifically active, which may contribute to its greater susceptibility to overexpression in aggressive cancers (Redis et al., 2016). Compared with the distribution of KGA in the cytoplasm, GAC is located in mitochondria with a lower Km-app, maintaining mitochondrial GLS metabolism in tumor cells (Cassago et al., 2012). GLS1 expression is regulated by a variety of factors, such as transcription factor c-Myc, transcription factor c-Jun, Sirtuin 5, NF-κB, and hypoxia-inducing factor 1 (HIF-1) (Gao et al., 2009; Rathore et al., 2012; Lukey et al., 2016; Greene et al., 2019; Xiang et al., 2019). The GLS2 gene is located on human chromosome 12 and consists of the short-form liver glutaminase (LGA) and glutaminase B (GAB) (Katt et al., 2017). However, the concrete role of GLS2 in tumors remains unclear, although some studies showed that it may inhibit tumor growth in some cancers (Liu et al., 2014; Martín-Rufián et al., 2014). Unlike GLS2, GLS1 is often overexpressed in many types of cancer, including breast cancer, acute myeloid leukemia, and non-small cell lung cancer, making it a promising therapeutic target for cancer treatment (Van Den Heuvel et al., 2012; Gross et al., 2014; Jacque et al., 2015). The small molecule inhibitors or gene silencing of GLS1 expression has shown antitumor activity in a variety of cancers, including melanoma (Varghese et al., 2021), breast cancer (Gross et al., 2014), pancreatic cancer (Son et al., 2013), acute myeloid leukemia (Emadi et al., 2014), and malignant glioma (Yamashita et al., 2021). Until now, some inhibitors targeting GLS1, including DON, BPTES, Compound 968, and CB-839, have been investigated in the treatment of cancers. (Figure 2).

#### 3.1.1 BPTES

BPTES is a specific GLS1 inhibitor that forms a stable inactive tetrameric form of KGA (Katt et al., 2012; Thangavelu et al., 2014). BPTES binds to the conformational pocket at the dimer interface of KGA, leading to conformational changes in the key loop (Glu312-Pro329) near the catalytic site (Thangavelu et al., 2012; Ferreira et al., 2013; Thangavelu et al., 2014). The amino acid residues in the key loop contribute to stabilizing the active site and enzyme activity. This results in the inhibition of phosphorylation activation of GLS, resulting in an inactive tetramer (Thangavelu et al., 2012; Ferreira et al., 2013; Thangavelu et al., 2014). Unlike DON, BPTES can also inhibit phosphorylated GLS (Katt et al., 2012). In 2010, Seltzer et al. reported that it could significantly inhibit the proliferation of glioblastoma cells with isocitrate dehydrogenase 1 (IDH1) mutation (Seltzer et al., 2010). These tumor cells can not consume isocitrate to produce α-KG but convert α-KG to 2-hydroxyglutaric acid (2-HG). Because glutamine is the source of α-KG, glutaminase inhibitors such as BPTES interfere with α-KG homeostasis and lead to inhibited proliferation in cancer cells carrying this mutant IDH1 (Seltzer et al., 2010). In a mouse xenograft osteosarcoma model, BPTES reduced the tumor weight of chondrosarcomas with IDH1 mutations and promoted tumor cell apoptosis (Zhang et al., 2022). It could dramatically reduce the colony-forming ability of glutamine-dependent ovarian cancer cells (Masamha and LaFontaine, 2018). Furthermore, combining BPTES with chemotherapeutic drugs resensitizes drug-resistant cancer cell lines to chemotherapy, regardless of their glutamine dependence status (Masamha and LaFontaine, 2018). Due to the low structural similarity to glutamine, BPTES has low off-target properties and decreased side effects (Xiang et al., 2015; Zimmermann et al., 2016). However, recent studies have shown that BPTES treatment also upregulates programmed cell death 1 ligand 1 (PD-L1) expression, resulting in an immunosuppressive tumor microenvironment (Byun et al., 2020). In addition, low bioavailability, poor metabolic stability, and low solubility greatly limit its clinical application (Shukla et al., 2012; Chen and Cui, 2015).

#### 3.1.2 CB-839

CB-839 (Telaglenastat) is one of the most promising BPTES analog drugs with improved oral bioavailability and tumor suppression effect (Gross et al., 2014; Chen and Cui, 2015; Ramachandran et al., 2016). Therefore, CB-839 has been tested in many clinical trials, including colorectal cancer (NCT02861300), triple-negative breast cancer (NCT02071862), renal cell carcinoma (NCT03163667), melanoma (NCT02771626), and others (Biancur et al., 2017; Choi and Park, 2018) (Table 2). Due to the complexity and heterogeneity of tumor metabolism, glutaminase blockade alone often leads to tumor cell resistance (Biancur et al., 2017; Dagogo-Jack and Shaw, 2018; Dos Reis et al., 2019). Combining GLS1 inhibition with other drugs provides a promising strategy for enhanced cancer treatment. Upregulated GLS1 expression in TNBC cell lines contributed to poor overall survival. CB-839 exhibits increased cytotoxicity against cancer cell lines with high-GLS1 expression (such as HCC 1806) by decreasing GSH production (Hong et al., 2022). It can also enhance cancer cells’s sensitivity to cisplatin and prolong the survival of mice. In MDA-MB-468 cells with low GLS1 expression, combined CB-839 with cisplatin does not show a significant synergistic antitumor effect (Hong et al., 2022). In TNBC xenograft models and basal-like breast cancer, CB-839, either alone or in combination with paclitaxel, exhibits a potent antitumor effect (Gross et al., 2014). Furthermore, a clinical phase II trial (NCT03057600) is conducted to evaluate the combination of CB-839 and paclitaxel to treat TNBC (Shen et al., 2021). In TNBC cells with decreased GLS expression, increased levels of carnitine palmitoyltransferase 1A/B (CPT1A/B), CPT2, and carnitine O-acetyltransferase (CRAT) may be potential predictive markers for CB-839 resistance. It is worth noting that inhibiting glutaminase and CPT1 can reduce the proliferation and migration of resistant cells, providing a potential therapy for TNBC (Dos Reis et al., 2019). In a preclinical melanoma model, CB-839 significantly improved the therapeutic efficacy of adoptive T-cell therapy and immune checkpoint inhibitors (anti-PD1 and anti-CTLA4), triggering a superior immune response (Varghese et al., 2021). A phase II clinical trial (NCT03163667) indicated that CB-839 and mTOR inhibitor (everolimus) showed good tolerability and improved progression-free survival in patients when treating advanced clear cell renal cell carcinoma (ccRCC) (Lee et al., 2022). In the previous phase I experiment, CB-839 combined with cabozantinib demonstrated good safety and efficacy in the treatment of metastatic renal cell carcinoma (RCC) (Tannir et al., 2018; Meric-Bernstam et al., 2022). In another randomized clinical trial, CB-839 did not enhance the therapeutic efficacy of cabozantinib in metastatic RCC (Tannir et al., 2021). This discrepancy may be caused by the poor inhibition of VEGFR inhibition on glucose metabolism or the lack of biomarkers to predict GLS inhibitory response in tumor cells. In colorectal cancer models, the combination of CB-839 and cetuximab significantly inhibited cell proliferation and increased apoptosis and necrosis, accompanied by decreased levels of the mTOR signaling target pS6, resulting in delayed tumor growth. The combination strategy demonstrated the effectiveness of CB-839 treatment in cetuximab-sensitive and cetuximab-resistant models (Cohen et al., 2020). Metformin, a commonly used anti-diabetic drug, has been shown to alter tumor cell metabolism and exert antitumor effects in recent years (Lu et al., 2021; Allende-Vega et al., 2022). Ren et al. demonstrated that combining CB-839 and metformin prevents compensation for metformin-induced electron transport chain inhibition through increased glutamine consumption to produce intermediates for cell proliferation, significantly inhibiting osteosarcoma growth and metastasis (Ren et al., 2020). This combination formulation has proved effective in other tumors, such as esophageal squamous cell carcinoma (Qie et al., 2019). In addition, CB-839 has been extensively studied in hematological malignancies such as acute myeloid leukemia (AML) (Gregory et al., 2019), myelodysplastic syndrome (MDS) (Okabe et al., 2022), and multiple myeloma (MM) (Das et al., 2014).

**TABLE 2:** Clinical trials on targeting glutamine metabolism.

Trial number	Treatment	Indication	Outcome	Phase	Status (Number of patients)	Reference
NCT02071927	CB-839 or CB-839+ azacitidine	Acute myeloid leukemia and acute lymphocytic leukemia	CB-839 was well tolerated and produced strong GLS inhibition in platelets and tumors	I	Completed (43)	Wang et al. (2015b)
NCT02071888	CB-839 or CB-839+ dexamethasone or CB-839+ pomalidomide + dexamethasone	Hematological tumors	CB-839 was well tolerated and produced strong GLS inhibition in platelets and tumors	I/II	Completed (25)	Vogl et al. (2015)
NCT02071862	CB-839 or CB-839+ standard chemotherapy (paclitaxel/everolimus/erlotinib/docetaxel/cabozantinib)	Solid tumors (TNBC, NSCLC, RCC, etc.)	CB-839 was well tolerated, caused significant glutaminase inhibition, and showed initial clinical activity against multiple tumor types	I	Completed (210)	Harding et al. (2015)
NCT03047993	CB-839+ azacitidine	MDS	Combination therapy is safe and effective. They responded well in previously treated and high-risk patients with MDS. CB-839 showed *in vivo* activity against leukemia stem cells	I/II	Completed (29)	Saxena et al. (2020)
NCT03163667	CB-839+ everolimus (CBE) or Placebo + everolimus (PhoE)	ccRCC	The CBE group was well tolerated and the median progression-free survival (3.8 months) was better than that of PhoE (1.9 months)	II	Completed (69)	Lee et al. (2022)
NCT03057600	CB-839+paclitaxel	TNBC	The combination treatment was well tolerated. The objective response rate (ORR) of metastatic TNBC patients without prior systemic treatment was 41%. The ORR of metastatic TNBC patients with ≥2 previous systemic therapies (including taxane) was 21%	II	Completed (52)	Vidal et al. (2019)
NCT03428217	CB-839 + cabozantinib (CB-Cabo) or Placebo + cabozantinib (Pbo-Cabo)	RCC	CB-Cabo was well tolerated, but CB-839 did not improve the efficacy of cabozantinib	II	Completed (444)	Tannir et al. (2021)
NCT03965845	CB-839 + palbociclib	Solid tumors with K-Ras mutation	NA	I/II	Completed (53)	NA
NCT03831932	CB-839 HCl + osimertinib	NSCLC with EGFR mutation	In progress	I/II	Active-Recruiting (24)	NA
NCT02861300	CB-839 + capecitabine	Solid tumors and fluoropyrimidine-resistant PIK3CA mutant colorectal cancer	CRC patients with PIK3CA mutations have a prolonged progression-free survival. The trial continues	I/II	Active-No recruiting (53)	Eads et al. (2018)
NCT03798678	CB-839 HCl + carfilzomib + dexamethasone	MM	The combination treatment was well tolerated. The trial continues	I	Active-No recruiting (36)	Gonsalves et al. (2022)
NCT03263429	CB-839 + panitumumab + irinotecan (Phase I)	metastatic and refractory RAS wildtype colorectal cancer	The triple therapy was well tolerated. CB-389 and panitumumab have shown some efficacy in heavily pretreated patients	I/II	Active-No recruiting (29)	Ciombor et al. (2019), Ciombor et al. (2023)
	CB-839 + panitumumab (Phase II)					
NCT03528642	CB-839 HCl + radiation therapy + temozolomide	IDH-mutated diffuse or anaplastic astrocytoma	In progress	I	Active-No recruiting (40)	NA
NCT03872427	CB-839 HCl	Specific pathway aberrant tumors (MPNST, NF1, KEAP1/NRF2, STK11/LKB1)	No dose-limiting toxicities were observed. The trial continues	II	Active-No recruiting (108)	Kizilbash et al. (2022)
NCT05521997	CB-839 + radiation treatment + cisplatin	Advanced cervical cancer	NA	II	Not yet recruiting	NA
NCT03875313	CB-839 + talazoparib	Solid Tumors (ccRCC, TNBC, colorectal cancer)	Slow enrollment	I/II	Terminated	NA
NCT03944902	CB-839 + niraparib	Platinum-resistant BRCA wild-type ovarian cancer	Company choosing not to continue with drug. One participant is now off the study	I	Terminated	NA
NCT02771626	CB-839 + nivolumab	ccRCC, melanoma, NSCLC	Lack of efficacy	I/II	Terminated	NA
NCT04265534	CB-839 (or placebo) + pembrolizumab + standard chemotherapy (carboplatin/pemetrexed)	NSCLC (KEAP1/NRF2-mutated, stage IV, nonsquamous)	Lack of clinical benefit	II	Terminated	NA
NCT04250545	CB-839 HCl + sapanisertib	Advanced NSCLC	Drug supply issues	I	Suspend	NA
NCT04824937	CB-839 + Talazoparib	Prostate cancer	NA	II	unknown status	NA
NCT02891538	EGCG	Chemoprevention in patients with radical resection of colorectal cancer	NA	I	Active-Recruiting (50)	NA
NCT06027086	DRP-104 + durvalumab	Advanced-stage fibrolamellar carcinoma	In progress	I/II	Not yet recruiting	NA
NCT04471415	DRP-104 or DRP-104 + atezolizumab	Advanced solid tumor	The company decided to close the study and discontinue further patient enrollment	I/II	Terminated	NA
NCT03894540	IPN60090 or IPN60090 + pembrolizumab/paclitaxel	Advanced solid tumor	The company decided to discontinue the study, but not due to any safety or tolerability concerns with IPN60090	I	Terminated	NA

#### 3.1.3 DON

DON (6-diazo-5-oxo-l-norleucine), a glutamine analog, irreversibly inhibits various glutamine utilization enzymes, such as glutaminase and glutamine synthetase, by forming covalent bonds with nucleophilic residues on the active site of the enzyme (Rajagopalan and DeBerardinis, 2011; Thomas et al., 2013; Gao et al., 2021). In the catalytic hydrolysis of glutamine, Ser286 at the active site of the GLS1 enzyme carries out a nucleophilic attack on the acyl carbon of glutamine, and the subsequent proton transfer of Tyr466 promotes the hydrolysis reaction. Ser286 performs the same nucleophilic attack on DON, but then releases diazo groups and leads to the formation of enzyme-inhibitor complexes (Thangavelu et al., 2014). Until now, DON has been utilized in numerous clinical trials for treating different cancers (Lyons et al., 1990; Cervantes-Madrid et al., 2015). Despite good antitumor activity, DON has multiple targets and some side effects such as nausea and vomiting (Cervantes-Madrid et al., 2015; Kulkarni et al., 2017). Due to caused severe gastrointestinal toxicity, many studies have been terminated (Magill et al., 1957; Ovejera et al., 1979; Ahluwalia et al., 1990). Similar to DON, acivicin, a glutamine analog, has been proven to inhibit tumor growth in many cancers. Acivicin has severe side effects in some clinical trials, such as central nervous system damage, bone marrow suppression, and gastrointestinal toxicity (Adolphson et al., 1986; Bonomi et al., 1994; Hidalgo et al., 1998; Mena et al., 2007). It was reported that broad-spectrum glutamine antagonists are more effective in promoting tumor regression than selectively inhibiting a single glutaminase (Hensley et al., 2013). Therefore, DON treatment can not only reduce the self-renewal potential and metastatic ability of pancreatic ductal adenocarcinoma (PDAC) tumor cells but also lead to extensive remodeling of the tumor extracellular matrix (ECM), promoting the infiltration and activation of CD8^+^ T cells (Sharma et al., 2020). Moreover, DON treatment sensitizes pancreatic tumors to immune checkpoint blockade therapy, resulting in tumor growth inhibition and prolonged survival (Sharma et al., 2020).

To improve biosafety, researchers have developed a series of stable prodrugs preferentially released DON in the tumor microenvironment (Lemberg et al., 2018; Tenora et al., 2019; Gao et al., 2021). Lemberg et al. showed that DON prodrug which was daily administrated at a low dose significantly inhibited the growth of EL-4 lymphoma in mice without obvious body weight changes (Lemberg et al., 2018). Lukáš Tenora et al. developed substituted acetylated lysine prodrugs to improve the delivery efficiency of DON to tumors (Tenora et al., 2019). The prodrug was stable in normal tissues but can be easily hydrolyzed into DON in P493B lymphoma cells, inhibiting tumor cell proliferation in a dose-dependent manner (Tenora et al., 2019). Recently, a new DON prodrug JHU395, has been developed to treat medulloblastoma (Pham et al., 2021). It can inhibit the growth of medulloblastoma cells at a low concentration. More importantly, due to better hydrophobicity than DON and JHU083, JHU395 allows for better penetration into the brain and effectively kills MYC-expressing medulloblastoma cells with decreased peripheral exposure. JHU395 can prolong the median survival of mice bearing human MYC-amplified medulloblastoma xenografts from 26 to 45 days (Pham et al., 2021). It also demonstrated good therapeutic effects in treating malignant peripheral nerve sheath tumors (MPNST) (Lemberg et al., 2020).

JHU083, another novel DON prodrug developed in recent years, has shown a remarkable tumor growth inhibition effect in various tumors (Hanaford et al., 2019; Leone et al., 2019; Yamashita et al., 2021; Huang et al., 2022). JHU083 is synthesized by decorating leucine amide and ethyl ester on the amino and carboxylic groups of DON. In TME, DON is produced after enzymatic cleavage of JHU083 (Hanaford et al., 2019). JHU083 can alter tumor metabolism and reprogram the immunosuppressive tumor microenvironment, leading to long-lasting immune memory for immunotherapy. Oral administration of JHU083 could penetrate brain tissue and significantly improve the survival rate of various MYC-driven medulloblastoma mouse models (Hanaford et al., 2019). Therefore, reduced gastrointestinal exposure enhanced its clinical biosafety. Combining JHU083 and programmed cell death protein 1 (PD-1) inhibitors produces better antitumor effects than PD-1 inhibitors alone (Leone et al., 2019). Furthermore, researchers have shown that glutamine antagonists can inhibit cancer cell oxidative and glycolytic metabolism, thereby reducing tumor hypoxia, acidosis, and nutrient consumption. Upregulated oxidative metabolism makes effector T cells a long-lived, highly activated phenotype. This difference in metabolism between tumor cells and T cells provides promising targets for the development of new metabolic antagonists (Leone et al., 2019). JHU083 has also demonstrated excellent therapeutic efficacy in preclinical models of IDH-mutant gliomas and significantly reduces mTOR signaling (Yamashita et al., 2021). A study has shown that JHU083 can substantially inhibit the production and recruitment of myeloid-derived suppressor cells (MDSCs) and induce the polarizing tumor-associated macrophages (TAMs) into pro-inflammatory phenotype. JHU083 also inhibits the expression of indoleamine 2,3-dioxygenase (IDO) in tumor cells, leading to decreased kynurenine levels and delayed tumor growth and metastasis by enhancing antitumor immune response (Oh et al., 2020). Huang et al. demonstrated that combining JHU083 and an epidermal growth factor receptor (EGFR) peptide vaccine (EVax) inhibits tumor growth in a mouse lung cancer model. JHU083 treatment reduces immunosuppressive MDSCs and converts TAM into pro-inflammatory macrophages (Huang et al., 2022).

Recently, a new DON prodrug, DRP-104 (sirpiglenastat) was developed, which carries isopropyl ester and acetylated tryptophan on the C-terminal and N-terminal of DON (Rais et al., 2022). DRP-104 is inactivated as an inert metabolite in gastrointestinal tissue, but it is activated at tumor sites and releases DON for therapeutic effect. DRP-104 can promote apoptosis of tumor cells, and activate innate and adaptive immune response *in vivo*, such as inducing polarization of TAM to M1 phenotype, reducing T cell depletion, and increasing NK cells (Yokoyama et al., 2022). The immune regulation of DRP-104 showed great potential for combined use of DRP-104 with immunotherapy. In 2020, combined DRP-104 with atezolizumab (anti-PD-1) entered the clinical trial (NCT04471415) in the treatment of advanced solid tumors (Johnson et al., 2021). A study has shown that DRP-104 can significantly inhibit the tumor growth of a variety of PDAC models *in vivo* (Encarnación-Rosado et al., 2023). DRP-104 treatment contributed to the compensatory increase of ERK signal, activating survival signal pathways in tumor cells to combat metabolic stress. The combination therapy of ERK kinase inhibitor trimetinib and DRP-104 significantly increased the survival rate of the homogeneous PDAC model (Encarnación-Rosado et al., 2023). In addition, DRP-104 also showed good antitumor activity in prostate cancer and lung cancer (Moon et al., 2023; Pillai et al., 2023).

#### 3.1.4 Compound 968

Compound 968 is a specific allosteric inhibitor of GAC (Wang et al., 2010). It does not disrupt the interaction between dimers in the glutaminase tetramer but binds to the hydrophobic pocket between the monomer-monomer interface of the GAC, located at the junction where the N-terminal and C-terminal of the enzyme meet the catalytic domain, thereby preventing the GAC from activating conformational changes (Wilson et al., 2013; Stalnecker et al., 2015). However, it only inhibits the inactive form of GAC and has no inhibitory effect on the activated form (Katt and Cerione, 2014). Compound 968 was first discovered as an inhibitor of Rho GTPase conversion. Compound 968 can inhibit Rho GTPase-induced carcinogenic transformation by targeting glutaminase activity in some transformed and cancer cells with Rho GTPase/NF-κB-dependent GLS activation (Wang et al., 2010; Wilson et al., 2013). Yuan et al. found that compound 968 can significantly induce cell cycle arrest and apoptosis and inhibit the proliferation of ovarian cancer cells (Yuan et al., 2016). Compound 968 can not only effectively inhibit the proliferation of cancer cells (Katt et al., 2012; Yuan et al., 2016), but also enhance the sensitivity of cancer cells to chemotherapy drugs or metabolic signaling inhibitors (Xie et al., 2016; Wang et al., 2021; Guo et al., 2023). Combined compound 968 with paclitaxel demonstrated an enhanced tumor-killing effect on cancer cells. In addition, Compound 968 treatment suppresses the AKT/mTOR/S6 signaling pathway in endometrial cancer cells (Guo et al., 2023). In addition, inhibition of GLS has been reported to increase the immunotherapy efficacy of CD8^+^ T cells (Johnson et al., 2018; Nabe et al., 2018). Compound 968 can enhance the secretion of CXCL10 and CXCL11 in ovarian cancer cells, and promote T-cell infiltration into tumors. Combining compound 968 and anti-PD-L1 antibody enhances the secretion of granzyme B from T cells and improves the antitumor activity of CD8^+^ T cells, prolonging the overall survival of ovarian cancer mice. Combining two drugs shows significantly better antitumor efficacy than monotherapy (Wang et al., 2021). Until now, most research based on glutaminase inhibitors for cancer treatment mainly focuses on GLS1, GLS2 is considered a tumor suppressor in glioblastoma and liver cancer (Matés et al., 2018). However, upregulated GLS2 plays an important role in the luminal subtype of breast cancer. Compound 968 can inhibit the activity of GLS2 and overcome the resistance of cancer cells to selective GLS1 inhibitors, resulting in tumor suppression (Lukey et al., 2019).

### 3.2 Glutamine uptake inhibitors

Cancer cells consume large amounts of glutamine to meet their energy demands for rapid proliferation (Wise and Thompson, 2010; Carvalho et al., 2019). Due to the hydrophilic property, extracellular glutamine cannot pass through the cell membrane and relies on glutamine transporters on the cell membrane (Li et al., 2021). To date, four glutamine transporter gene families have been identified, namely, SLC1, SLC6, SLC7, and SLC38, encompassing a collective count of 14 members (Bhutia and Ganapathy, 2016). Among these members, ASCT2 (alanine-serine-cysteine transporter 2) is considered to be the most important glutamine transporter in tumor cells, which is encoded by the SLC1A5 gene, is a Na^+^ dependent neutral amino acid transporter (Scalise et al., 2018; Zhang et al., 2020). ASCT2 (SLCA15) can transport glutamine, alanine, serine, and threonine into the interior of cells, and induce the efflux of glutamine, serine, threonine, and asparagine (Jiang et al., 2020; Scalise et al., 2022). ASCT2 can not transport cysteine, which acts as a regulator to induce glutamine efflux (Scalise et al., 2015). ASCT2 is a homologous trimer, with each monomer typically consisting of eight transmembrane domains and two hairpin rings (HP), consisting of a scaffold domain and a transport domain, respectively. The scaffold domain provides the trimer interface, and the transport domain is used for binding amino acids and coupling ions. During the process of transport, the two hairpin rings move like an elevator lift, mediating the transport of amino acids from the outside of the cell to the binding site (Garaeva et al., 2019; Lopes et al., 2021). Many factors are involved in regulating ASCT2 expression, including the transcription factors c-Myc, RNF5, Leptin, and Insulin (Fanjul et al., 2012; Jeon et al., 2015; Van Geldermalsen et al., 2016). In breast cancer, RNF5 loss is associated with increased ASCT2 expression, finally leading to poor patient outcomes, and poor response to paclitaxel (Jeon et al., 2015). ASCT2 is overexpressed on the surface of various tumor cells, such as gastric cancer (Ma et al., 2021), colon cancer (Ma et al., 2018), pancreatic cancer (Wang et al., 2022), and TNBC (Van Geldermalsen et al., 2016). Small molecule inhibitors targeting ASCT2 and other glutamine transporters have been shown to inhibit glutamine uptake and consumption and exhibit antitumor effects in various cancer models.

Besides ASCT2 for glutamine transportation, SLC38A1 (SNAT1) and SLC38A2 (SNAT2) can also drive the influx of glutamine into cells (Jin et al., 2023). Although SLC38A1 and SLC38A2 have broad substrate specificity, they have a higher affinity for glutamine compared to other transporters (Pochini et al., 2014). SLC38A1 mainly participates in the transport of glutamine in tumor cells in the absence of ASCT2 (Bröer et al., 2016). In addition, SLC38A1overexpression can increase the phosphorylation levels of Akt and mTOR in osteosarcoma, breast cancer, and HCC (Böhme-Schäfer et al., 2022). SLC38A2 not only provides glutamine for energy metabolism but also contributes to the accumulation of leucine and proline, which activate the mTORC1 signal and participate in tumor cell metabolism and epigenetic control (Menchini and Chaudhry, 2019; Patriarca et al., 2021). SLC38A1 is overexpressed in various tumors such as melanoma (Böhme-Schäfer et al., 2022), gastric cancer (Xie et al., 2014), acute myeloid leukemia (Li Y. et al., 2019), and liver cancer (Liu et al., 2021). SLC38A2, which is overexpressed in TNBC (Morotti et al., 2021), gastric cancer (Zhu et al., 2023), pancreatic cancer (Parker et al., 2020), and other tumors, is involved in tumor growth, and metastasis. Inhibition of SLC38A1 through siRNA-mediated gene silencing or N-methyl-aminoisobutyric acid (MeAIB) can reduce the growth and metastasis of various tumors (Bröer et al., 2016; Park et al., 2016; Böhme-Schäfer et al., 2022).

SLC6A14 (also known as ATB^0,+^) is a Na^+^/Cl^−^ coupled transporter protein that maintains a single amino acid coupled with two Na^+^ and one Cl^−^ ions flowing unidirectionally into cells (Sloan and Mager, 1999). SLC6A14 relies on the ion gradient on the plasma membrane to drive the intracellular accumulation of amino acids, even under unfavorable amino acid concentration gradients (Nakanishi et al., 2001). It can transport 18 amino acids that make up proteins, including glutamine (Karunakaran et al., 2011; Lu et al., 2022). SLC6A14 participates in glutamine addiction in tumor cells and other amino acid-related tumor metabolic pathways. For example, it can help tumor cells take up methionine to maintain DNA methylation and mediate the transport of carnitine (Nakanishi et al., 2001; Sniegowski et al., 2021). SLC6A14 is overexpressed in colorectal cancer (Lu et al., 2022), ER^+^ breast cancer (Karunakaran et al., 2011), gastric cancer (Wang and Wang, 2022), pancreatic cancer (Schniers et al., 2022), and cervical cancer (Sikder et al., 2018), closely related to lower overall survival. α-Methyltryptophan (α-MT), a pharmacological inhibitor of SLC6A14, can inhibit the proliferation of cancer cells *in vitro* and tumor growth *in vivo* (Karunakaran et al., 2011; Coothankandaswamy et al., 2016; Lu et al., 2022).

**FIGURE 3:**
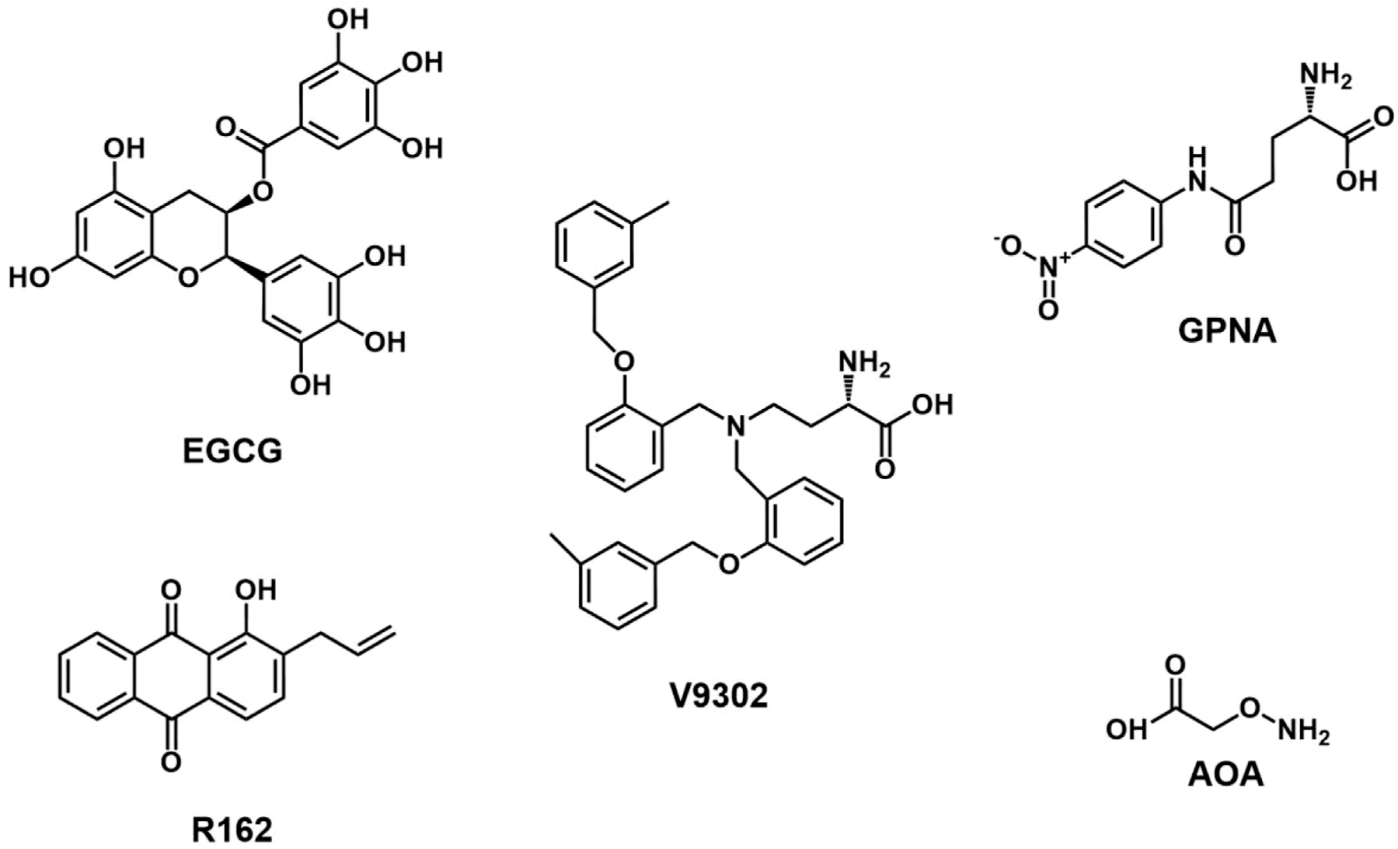
Chemical structure of inhibitors targeting glutamine uptake and transformation.

After catalysis by GLS in cells, glutamine is converted into glutamate. The generated glutamate can directly participate in the synthesis of glutathione, or generate cysteine which is another substrate of glutathione synthesis through the coupled action of glutamate efflux mediated by SLC7A11 and cysteine uptake (Timmerman et al., 2013). Study have shown that SLC7A11 reduced the therapeutic effect of ROS-type drugs by reversing oxidative stress, thereby causing tumor chemotherapy resistance (Huang et al., 2005). In addition, recent studies have found that high expression of SLC7A11 promotes the development of tumors by inhibiting ferroptosis of tumor cells (Dixon et al., 2012; Zhang et al., 2018). Therefore, inhibiting SLC7A11 can interfere with the synthesis of glutathione in tumor cells and disrupt the oxidative-reductive homeostasis of cancer cells (Bhutia et al., 2015; Zhang et al., 2018). Common SLC7A11 inhibitors including sulfasalazine, erastin, sorafenib, and IKE, have shown good antitumor effects in various cancer models (Bhutia et al., 2015; Yang J. et al., 2021; Li H. et al., 2022). A recent study showed that high expression of SLC7A11 increases the sensitivity of tumor cells to oxidative stress and promotes the growth of primary tumors (Yan et al., 2023). Despite the reported various glutamine transporters, until now, ASCT2 is still the most extensively studied transporter in cancer cells. Therefore, inhibitors targeting ASCT2 were discussed in this study (Figure 3).

#### 3.2.1 γ-L-glutamyl-4-nitroanilide (GPNA)

GPNA, a glutamine analog synthesized by Esslinger, has been widely used to inhibit the glutamine transporter ASCT2 (Esslinger et al., 2005). GPNA can inhibit ASCT2 and other glutamine transporters, such as sodium-coupled neutral amino acid transporter 1 (SNAT1), SNAT2, large neutral amino acid transporter 1 (LAT1), and LAT2, leading to decreased uptake of essential amino acids (Bröer et al., 2016; Chiu et al., 2017). The antitumor activity of GPNA may also be attributed to the interaction with excitatory amino acid transporters (EAATs) and substantial disruption of intracellular chloride or water homeostasis (Freidman et al., 2022). Corti et al. indicated that GPNA can be hydrolyzed by γ-glutamyltransferase (GGT) to produce p-nitroaniline (PNA), reducing the viability of lung cancer A549 cells without decreased cellular glutamine uptake (Corti et al., 2019). It is unclear whether the delayed cell growth after GPNA treatment is caused by ASCT2 inhibition. Ma et al. demonstrated that blocking ASCT2 with GPNA significantly enhanced the anti-proliferative effect of trastuzumab in gastric cancer *in vitro* and *in vivo* (Ma et al., 2021). GPNA treatment also inhibited cell growth in pancreatic cancer (Wang et al., 2022). In triple-negative basal-like breast cancer cells, GPNA significantly decreased glutamine uptake and inhibited the mTORC1 signaling pathway, leading to delayed tumor growth (Van Geldermalsen et al., 2016). Recent studies have shown that glutamine deprivation can inhibit cholesterol synthesis in hepatocellular carcinoma (HCC) by inducing lipid autophagy, thereby reducing NADPH consumption during cholesterol synthesis and maintaining redox balance. The re-activation of cholesterol synthesis further enhances the antitumor effect induced by GPNA *in vivo* (Kong et al., 2023).

#### 3.2.2 V-9302

In 2018, Charles Manning et al. reported a competitive small molecule antagonist V-9302, which selectively targeted the ASCT2 transporter for glutamine influx inhibition (Schulte et al., 2018). V-9302 treatment can kill tumor cells by increasing oxidative stress and inhibiting the proliferation of colon cancer cells (Schulte et al., 2018). However, subsequent studies have shown that V-9302 also blocks SNAT2 (SLC38A2) and LAT1 (SLC7A5) in 143B osteosarcoma cells and HCC1806 breast cancer cells, suggesting potential off-target effects (Bröer et al., 2018). ASCT2 gene silencing contributes to the upregulated expression of other glutamine transporters, such as SNAT1 and SNAT2 (Bröer et al., 2016; Bröer et al., 2019). Therefore, simultaneously targeting ASCT2, SNAT1, and SNAT2 may be a potential strategy to inhibit glutamine influx. Zhang et al. demonstrated that V-9302 treatment inhibited ASCT2 expression and decreased the intracellular levels of glutamine and glutathione, promoting apoptosis, autophagy, and oxidative stress (Zhang et al., 2020). V-9302 also resensitized paclitaxel-resistant ovarian cancer cells to paclitaxel by downregulating the mTORC1/S6K signaling pathway by increased phosphorylation of Bcl-2 and decreased levels of Mcl-1 (Kim et al., 2022). Recently, V-9302 has been shown to decrease B7H3 expression (T cell coinhibitory molecule) on tumor cells through ROS-mediated autophagic degradation pathways and enhance CD8^+^ T cell infiltration and activation. Additionally, the combination treatment of V-9302 and anti-PD-1 antibody can improve antitumor immunity in a mouse breast cancer model, transforming “cold tumors” into “hot tumors” (Li Q. et al., 2022). V-9302 selectively inhibited the glutamine uptake by tumor cells in a spontaneous mouse TNBC model. It did not affect T cell uptake due to compensatory upregulation of the glutamine transporter SLC6A14 in CD8^+^ T cells, which increased CD8^+^ T cell activation and glutathione synthesis (Edwards et al., 2021). Byun et al. demonstrated that inhibiting glutamine metabolism in tumor cells can reduce the level of GSH, increase the expression of PD-L1 on the surface of tumor cells by damaging SERCA activity, and increase T cell-mediated cell death induced by Fas/Fasl signal (Byun et al., 2020). These studies show that V-9302 has a therapeutic effect in tumor cells by reducing the GSH level and mTOR activity and inducing autophagy, oxidative stress, and immune response. In addition, V-9302 and GLS inhibitor CB-839 combination therapy show significant tumor suppression in an HCC xenograft mouse model (Jin et al., 2020). In glutamine-addicted squamous cell carcinoma (SCC) cancer cells, V-9302 treatment inhibits the YAP1/TEAD signaling pathway to weaken the SCC cancer phenotype (Mickle, 2022). MLN4924, a small molecule inhibitor of neddylation, increases glutamine uptake in breast cancer cells by inactivating the CRL3-SPOP E3 ligase, leading to the ASCT2 upregulation. Inhibiting ASCT2 by V-9302 enhances the therapeutic effect of MLN4924 on tumor growth (Zhou et al., 2022). Despite good anti-tumor effects in some cancer models, V-9302 has poor water solubility and an off-target effect. Its interference with glutamine metabolism will also lead to compensatory enhancement of glucose metabolism in tumor cells (Luo et al., 2020).

#### 3.2.3 Benzylserine/benzylcysteine derivatives

Benzylserine/benzylcysteine derivatives can compete with neutral amino acids for the binding site of ASCT2 (Grewer and Grabsch, 2004). An oocyte uptake assay has shown that benzylserine can inhibit LAT1/2, ASCT2, and SNAT1/2 (van Geldermalsen et al., 2018). LAT1, which is often overexpressed in tumor cells, represents the main transporter for leucine uptake (Zhao et al., 2015). LAT1 inhibition has been shown to reduce tumor growth (Shindo et al., 2021; Cappoli et al., 2022; Kanai, 2022; Kurozumi et al., 2022). Benzylserine can reduce cell growth in melanoma and breast cancer by inhibiting leucine and glutamine cellular uptake (Wang et al., 2014; van Geldermalsen et al., 2018). However, developing inhibitors with higher affinity and specificity is needed due to the high dosage requirement and potential off-target effects.

#### 3.2.4 Monoclonal antibodies

In addition to small molecule inhibitors, monoclonal antibodies (mAbs) have high specificity and good stability targeting glutamine transporters (Zahavi and Weiner, 2020). Suzuki M et al. developed monoclonal antibodies (KM4008, KM4012, and KM4018) to bind with the extracellular domain of ASCT2 (Suzuki et al., 2017). Another monoclonal antibody, KM8094, can effectively inhibit ASCT2-mediated glutamine uptake and exert an antibody-dependent cell-mediated cytotoxicity (ADCC) effect (Osanai-Sasakawa et al., 2018). It could inhibit tumor growth and enhance docetaxel’s antitumor efficacy in a gastric cancer xenograft mouse model (Osanai-Sasakawa et al., 2018). Hara Y et al. developed Ab3-8, which does not directly inhibit ASCT2 but inhibits glutamine uptake by inducing the internalization of ASCT2 (Hara et al., 2020). It could inhibit the growth of KRAS mutant SW1116 and HCT116 human colon cancer cells (Hara et al., 2020). MEDI7247, an antibody-drug conjugate (ADC) that specifically binds to the extracellular domain of ASCT2, could release its payload pyrrolobenzodiazepine (PBD), promoting DNA damage and tumor cell death (Liu et al., 2018; Wei et al., 2023). MEDI7247 has shown remarkable antitumor activity in hematological malignancies such as AML, MM, diffuse large B cell lymphoma (DLBCL), acute lymphoblastic leukemia (ALL), Burkitt lymphoma, as well as solid tumors including colorectal cancer, small cell lung cancer, and head and neck squamous cell carcinoma (Pore et al., 2018; Schifferli et al., 2018; Wei et al., 2023). MEDI7247 entered phase I clinical trials for treating hematological malignancies (NCT03106428 and NCT03811652) (Hartley, 2021; Wei et al., 2023).

#### 3.2.5 Others

Researchers have made significant progress in developing various compounds as inhibitors of ASCT2-mediated glutamine uptake. Besides the compounds mentioned above, several other compounds have shown inhibitory activity on ASCT2. One notable compound is the proline analog γ-FBP, which is not a substrate of ASCT2. It could inhibit ASCT2-mediated glutamine uptake in human melanoma cells (Colas et al., 2015). Another proline analog, a benzylproline derivative, has also been identified as an inhibitor of ASCT2 (Singh et al., 2017). Additionally, lobetyolin has been found to induce apoptosis in colon cancer cells by inhibiting ASCT2 (He et al., 2020). Using a homology model of the outward conformation of ASCT2, researchers designed a novel inhibitor called L-cis-hydroxyproline benzoate (Lc-BPE). This inhibitor targets a stereo-specific pocket in the substrate binding site of ASCT2 and demonstrates submicromolar potency and stereo-selectivity. It has been shown to inhibit cell proliferation in various cancer cells, such as MCF-7, LnCaP, and MDA-MB-231 *in vitro* (Garibsingh et al., 2020).

IMD-0354 could disrupt the membrane localization of ASCT2 and attenuate mTOR signaling. The suppressed cell proliferation and induced cell cycle arrest collectively contribute to inhibited tumor growth in melanoma xenograft models (Feng et al., 2021). Topotecan (TPT), an inhibitor of DNA topoisomerase I, was recently discovered to downregulate ASCT2, leading to oxidative stress and apoptosis induction through the mitochondrial pathway in gastric cancer cells. The downregulated ASCT2 inhibited glutamine influx and demonstrated therapeutic potential for cancer treatment (Wang et al., 2019). Ag120 (ivosidenib) is a compound that targets tumor cells by inhibiting the production of 2-HG mediated by IDH1mt (Radoul et al., 2021). Ag120 has been shown to delay cancer cell proliferation and increase autophagy by inhibiting ASCT2-mediated glutamine metabolism (Yu et al., 2022). Mianserin, a tetracyclic antidepressant, has been shown to inhibit tumor growth in SW480 cells *in vitro* and *in vivo* through ASCT2 inhibition (Duan et al., 2022).

### 3.3 Glutamate dehydrogenase (GLUD/GDH) inhibitors

Glutamine is enzymatically converted to glutamate by glutaminase, and further conversion of glutamate to α-KG is facilitated by GLUD through oxidative deamination (Yoo et al., 2020). GLUD inhibitors can suppress tumor cell growth by hindering the replenishment of glutamine in the TCA (Hoerner et al., 2019; Yoo et al., 2020). Epigallocatechin gallate (EGCG), a bioactive compound derived from green tea (Eng et al., 2018), has been demonstrated to inhibit the formation of D-2-hydroxyglutarate (D-2-HG) from glutamate, augmenting the radiosensitivity in IDH mutant cancers (Peeters et al., 2019). Furthermore, EGCG has inhibitory effects on the proliferation and invasion of lung cancer cells *in vitro* (Jin et al., 2015). Combined with CB-839, EGCG synergistically inhibits the proliferation and promotes apoptosis of the KM3/BTZ multiple myeloma cell line by targeting glutamine metabolism and glycolysis (Li et al., 2023). However, it should be noted that EGCG exhibits low bioavailability and stability *in vivo* (Le et al., 2018; Kaur et al., 2019). Another key player in α-KG generation is the mitochondrial enzyme GDH1, which contributes to the accumulation of the subsequent metabolite fumarate. The activation of glutathione peroxidase enhances the clearance of ROS in cancer cells (Jin et al., 2015). Purpurin and its analog R162 disrupt the redox homeostasis of cancer cells by inhibiting GLUD. *In vitro*, R162 significantly inhibited the proliferation of lung cancer, breast cancer, and leukemia cell lines, but did not affect the normal proliferation of human cells (Jin et al., 2015). *In vivo* studies have shown that R162 prevents lung cancer metastasis in patient-derived xenograft models (Jin et al., 2018).

### 3.4 Aminotransferase inhibitors

Glutamate generated in mitochondria can also be catalyzed by glutamate oxaloacetate transaminase 2 (GOT2) and glutamate pyruvate transaminase 2 (GPT2) to form α- KG. Some studies have confirmed the important role of GOT2 and GPT2 in tumor cell growth. For example, GOT2 acetylation is essential to maintain the redox balance of pancreatic cancer cells (Yang et al., 2015). The expression level and activity of GOT2 and GPT2 in breast cancer cells were significantly higher than those in normal breast cells, which constituted the basis of glutamine addiction in breast cancer cells (Korangath et al., 2015). Aminooxy acetic acid (AOA) is a widely used inhibitor of aminotransferase that can inhibit tumor cell growth and apoptosis through induced endoplasmic reticulum stress (Korangath et al., 2015). In colon cancer, tumor cells with PIK3CA mutations can increase their dependence on glutamine for growth by upregulating GPT2. Inhibiting GPT2 with AOA can significantly delay the xenograft growth of PIK3CA-mutated colon cancer cells (Hao et al., 2016).

## 4 Challenges of targeted glutamine metabolism for Cancer treatment

Although targeting glutamine metabolism in cancer cells is a promising strategy, there are still many obstacles that need to be overcome to clinical outcomes.

### 4.1 Resistance to inhibitors of glutamine metabolism

Due to the plasticity of tumor cell metabolism, tumor cells evolved an adaptive metabolic network in the absence of glutamine supply. For example, when using GLS inhibitors to treat PDAC, metabolomic and proteomic analyses showed a significant increase in fatty acid oxidation-related metabolites and proteomics, suggesting a compensatory pathway for glutamine metabolism (Zhang et al., 2013; Biancur et al., 2017). When blocking glutamine metabolism, tumor cells can compensate by producing oxaloacetic acid through pyruvate carboxylase to maintain TCA cycle flux (Cheng et al., 2011). In CB-839-resistant TNBC cells, increased mitochondrial fatty acid β oxidation was observed (Dos Reis et al., 2019). These compensatory pathways can serve as promising targets for combination therapy. For example, the combination of 2-deoxyglucose (glycolytic inhibitor), metformin (glycolytic inhibitor), compound C (an AMPK inhibitor), MLN128 (mTOR1 inhibitor), and other glutamine metabolism inhibitors have shown synergistic antitumor effects in some cancer models (Elgogary et al., 2016; Momcilovic et al., 2018; Dos Reis et al., 2019; Luo et al., 2020). It is worth noting that when glutamine is depleted, upregulated amino acid transporters, such as SLC1A3 (aspartic acid/glutamate transporter) and SLC7A3 (arginine transporter) were observed. The arginine accumulation within tumor cells can activate mTORC1 signal transduction and aid in tumor growth and metabolic adaptation, while aspartic acid can participate in tumor cell nucleotide synthesis and TCA cycle (Tajan et al., 2018; Lowman et al., 2019). This suggests that blocking other amino acid transporters or consuming other key amino acids in tumor metabolism can help overcome resistance caused by glutamine blockade. In addition, the role of GLS2 in tumor cells has not been well studied. GLS2 expression is enhanced and associated with radiation therapy resistance (Xiang et al., 2013; Szeliga et al., 2014). Compound 968 has an inhibitory effect on GLS2 and can inhibit the growth of BPTES-resistant breast cancer (Lukey et al., 2019), suggesting the different roles of GLS2 in different tumors. These issues pose challenges in targeting GLS, therefore further research is needed to investigate the role of GLS2 in cancer treatment. The impact of targeted glutamine metabolism on anti-tumor immune response should not be ignored. Although BPTES and V-9302 treatment can promote M1 macrophage polarization or enhance CD8^+^ T cell anti-tumor function, they have also been found to enhance the expression of PD-L1 on tumor cells (Liu et al., 2017; Byun et al., 2020; Li Q. et al., 2022). These results suggest that glutamine metabolism inhibitors exert complex effects on anti-tumor immunity. The combination use of glutamine metabolism inhibitor and immune checkpoint inhibitor needs to be rationally designed (Yu et al., 2023). Finally, searching for biomarkers to predict the efficiency of glutamine targeting, or metabolomic and proteomic approaches to identify metabolic compensation mechanisms, will significantly improve the efficacy of cancer treatment.

### 4.2 Inherent deficiencies of glutamine metabolism inhibitors

Many reported glutamine metabolism inhibitors have shown serious off-target effects. For example, DON can extensively bind to glutamine-dependent enzymes, causing severe systemic toxicity in clinical trials (Kulkarni et al., 2017). In addition, some ASCT2 inhibitors can also block other amino acid transporters (SLC7A5, SLC38A2) (Bröer et al., 2018). The poor selectivity of inhibitors not only affects the therapeutic effect but also brings side effects on healthy tissues. BPTES and V-9302 have low solubility and low bioavailability, limiting their further application in tumor therapy (Tang et al., 2022; Yu et al., 2023). Developing efficient, and low-toxicity glutamine metabolism inhibitors for cancer treatment is greatly needed. A recent study has indicated that a microtubule inhibitor C118P can block ASCT2 function and contribute to apoptosis and G2/M cell cycle arrest. C118P showed improved efficacy and toxicity compared with GPNA and V-9302 (Lyu et al., 2023). A new GLS1 inhibitor compound 27 (IPN60090) was developed by replacing the flexible core of CB-839 with heterocycles. IPN60090 has shown a high oral exposure rate, good metabolic stability, and a strong inhibitory effect on GLS in preclinical studies (Soth et al., 2020). Secondly, prodrug strategies can efficiently deliver drugs to tumors with reduced side effects. Recently, Deng et al. developed a novel DON prodrug, HDON, which can selectively activate nitroreductase in hypoxic tumor environments without affecting oxygen-rich normal tissues, showing significant tumor inhibition in the H22 mouse liver cancer model (Zheng et al., 2024). Finally, developing smart drug delivery systems for glutamine metabolism inhibitors can help reduce toxic side effects and improve treatment outcomes (Shakeel, 2023). Researchers used Ce6 (photosensitizer), V-9302, and BMS-1 (PD-1/PD-L1 inhibitor) to develop a photodynamic immunostimulant BVC with good stability and high drug delivery efficiency. V-9302 in BVC nanoparticles can inhibit glutamine transport and GSH synthesis. A decrease in GSH levels can upregulate the expression levels of Fas and PD-L1 in tumor cells, and block the PD-1/PD-L1 pathway through BMS-1. On the other hand, Ce6-mediated photodynamic therapy induces immunogenic cell death in tumor cells, which further activates anti-tumor immunity (Zhao et al., 2023). Elham et al. loaded CB-839 into CD133 functionalized polyethylene glycol gold nanoparticles to treat glioblastoma. The glycoprotein antigen CD133 can target and inhibit glioblastoma stem cells (GSCs), while polyethylene glycol gold nanoparticles have good biocompatibility and can enhance the permeability of drugs to the blood-brain barrier (Poonaki et al., 2022). Luo et al. developed an oxidation-reduction sensitive micelle POEG-p-2DG based on 2-DG (a glycolytic inhibitor) prodrug, which can serve as a carrier for V-9302. The prodrug micelles loaded with V-9302 improved the bioavailability of V-9302 and demonstrated excellent anti-tumor activity by synergistically targeting two metabolic pathways (Luo et al., 2020).

## 5 Conclusion and future perspective

Targeting glutamine metabolism for cancer treatment has gained considerable attention in recent years. Inhibiting glutamine metabolism holds great potential to suppress tumor growth and improve treatment outcomes. The development of more potent and selective glutaminase inhibitors and effective delivery strategies remains an active area of research. However, there are several challenges for further application in treating advanced cancers. Firstly, considering the high tumor heterogeneity, various tumors or different regions within the same tumor can exhibit distinct metabolic profiles (Hensley et al., 2016). Therefore, developing universal antitumor drugs that inhibit glutamine metabolism for cancer treatment still represents a significant challenge. Secondly, the side effects remain another challenge to improve the antitumor efficacy of drugs targeting glutamine metabolism. Targeting glutamine metabolism inhibitors to tumor tissues but not healthy cells may circumvent these drawbacks. Developing smart delivery platforms for efficient glutamine metabolism disruption can minimize toxicity and improve therapeutic effects (Zhao et al., 2022). Furthermore, the study of biomarkers that predict tumor cell dependence on glutamine will improve the efficacy of glutamine inhibitors. Finally, the potential drug resistance may hinder the application of glutamine metabolism inhibitors for cancer treatment. The cancer cells may develop alternative metabolic pathways to compensate for the limited glutamine supply (Yang W.-H. et al., 2021). Revealing the drug resistance mechanisms and combination strategy will be essential to overcome resistance and improve treatment efficacy.

Despite these challenges, developing more potent and selective glutaminase inhibitors and smart delivery systems remains a cutting-edge research area. Effectively disrupting glutamine metabolism could open a new era for targeted cancer therapy. Furthermore, combination therapies that target multiple metabolic pathways, including glutamine metabolism, may enhance treatment outcomes and overcome resistance. Further research and clinical trials are greatly needed to exert the full potential of drugs targeting glutamine metabolism in cancer treatment.
